# Use of AI in Mental Health Care: Community and Mental Health Professionals Survey

**DOI:** 10.2196/60589

**Published:** 2024-10-11

**Authors:** Shane Cross, Imogen Bell, Jennifer Nicholas, Lee Valentine, Shaminka Mangelsdorf, Simon Baker, Nick Titov, Mario Alvarez-Jimenez

**Affiliations:** 1 Orygen Digital, 35 Poplar Rd, Parkville, Melbourne, 3052, Australia, 61 3 9966 9383; 2 Centre for Youth Mental Health, University of Melbourne, Melbourne, Australia; 3 School of Psychological Sciences, Macquarie University, Sydney, Australia; 4 MindSpot, Sydney, Australia

**Keywords:** mental health, health care, AI, community members, mental health professional, web-based survey, Australia, descriptive statistic, thematic analysis, cost reduction, data security, digital health, digital intervention, artificial intelligence

## Abstract

**Background:**

Artificial intelligence (AI) has been increasingly recognized as a potential solution to address mental health service challenges by automating tasks and providing new forms of support.

**Objective:**

This study is the first in a series which aims to estimate the current rates of AI technology use as well as perceived benefits, harms, and risks experienced by community members (CMs) and mental health professionals (MHPs).

**Methods:**

This study involved 2 web-based surveys conducted in Australia. The surveys collected data on demographics, technology comfort, attitudes toward AI, specific AI use cases, and experiences of benefits and harms from AI use. Descriptive statistics were calculated, and thematic analysis of open-ended responses were conducted.

**Results:**

The final sample consisted of 107 CMs and 86 MHPs. General attitudes toward AI varied, with CMs reporting neutral and MHPs reporting more positive attitudes. Regarding AI usage, 28% (30/108) of CMs used AI, primarily for quick support (18/30, 60%) and as a personal therapist (14/30, 47%). Among MHPs, 43% (37/86) used AI; mostly for research (24/37, 65%) and report writing (20/37, 54%). While the majority found AI to be generally beneficial (23/30, 77% of CMs and 34/37, 92% of MHPs), specific harms and concerns were experienced by 47% (14/30) of CMs and 51% (19/37) of MHPs. There was an equal mix of positive and negative sentiment toward the future of AI in mental health care in open feedback.

**Conclusions:**

Commercial AI tools are increasingly being used by CMs and MHPs. Respondents believe AI will offer future advantages for mental health care in terms of accessibility, cost reduction, personalization, and work efficiency. However, they were equally concerned about reducing human connection, ethics, privacy and regulation, medical errors, potential for misuse, and data security. Despite the immense potential, integration into mental health systems must be approached with caution, addressing legal and ethical concerns while developing safeguards to mitigate potential harms. Future surveys are planned to track use and acceptability of AI and associated issues over time.

## Introduction

Mental ill health is the leading cause of disability worldwide [1], yet fewer than half of all people with a mental health condition seek or receive evidence-based treatment [2-4]. Among the key structural barriers to effective care is that the demand outstrips the supply of qualified mental health professionals (MHPs), resulting in severely limited access and excessive wait times [5]. Moreover, MHPs are frequently burdened by substantial time-intensive administrative responsibilities and tasks, such as note-taking, detailed report writing, and planning for therapeutic sessions, limiting their availability to provide clinical care [6].

As digital technology becomes commonplace in society, tasks and services that were once performed manually, and often slowly, are now accomplished more efficiently via automated technology systems. However, despite many industries embracing new technologies for enhanced efficiency and responsiveness, the same progress has not been made in mental health care. Mental health care remains inaccessible, cumbersome to navigate, reactive, and slow to deliver, leaving mental health consumers frustrated and care providers burnt out [7].

People now have much greater access to information, including medical information and their own health data, than ever before [8]. In a contemporary landscape, where the prevalence of “on-demand” services is increasing, both mental health consumers and MHPs may expect comparable responsiveness. As a result, many are turning to digital products and services that aim to immediately address their needs. Young people, for example, are open and interested in using a range of digital technologies for mental health support, and many clinicians are already using these tools as part of routine care [9]. Wide-scale adoption of telehealth through the COVID-19 pandemic demonstrated the capacity for services to shift in response to changing demands, resulting in MHPs strongly endorsing the ongoing provision of technology-enhanced services [10]. This shift in attitude toward digital technology reflects an acknowledgment of the potential it holds for addressing barriers to providing effective and accessible care.

Recent advances in artificial intelligence (AI) have raised both excitement and debate concerning opportunities to harness this technology for mental health care [11,12]. AI encompasses a range of computer-based digital techniques and methodologies that perform cognitive processes characteristic of humans; such as learning, problem solving, pattern recognition, generalization, and predictive inference [13,14]. The recent advancement in natural language processing (NLP), a specialized branch of AI, has enabled chatbots and other language-driven systems to address requests, respond to queries, and provide advice autonomously, without human intervention [15]. Commercial tools such as ChatGPT enable users to enter any kind of query and obtain real-time responses significantly faster than traditional methods, such as internet searches.

A wide range of AI enabled products and services have been trialled in mental health care and health care more broadly [14]. AI has been used by health professionals to help solve complex problems such as identifying and diagnosing anomalies in medical images and genetic testing, predicting medical risk and disease prognosis, facilitating diagnostic and treatment decisions and recording and classifying clinical progress notes to name but a few [14]. For people with mental health difficulties, platforms such as Woebot [16] have been developed that use chatbots to deliver cognitive-behavioural therapy. More recently, tools such as ChatGPT have become freely available to the public, and with over 100 million users in its first few months, it was the fastest growing commercial application in history [17]. According to one community survey of over 1000 people in Australia, just under a half (48%) of Australians had heard of ChatGPT and almost a quarter (23%) had used it, with millennials (born between 1981 and 1996) and those with bachelor degrees and higher making up the majority of users [18]. Another youth survey found that 70% of young people 14‐17 have used ChatGPT, with 59% using it for study and 42% for completing school assignments [19].

Large language model technologies like ChatGPT, are increasingly used by certain groups of consumers as an alternative to seeing a qualified MHP, and by some MHPs to assist with burdensome administrative tasks [20]. A recent global survey of approximately 800 psychiatrists [21] found that 75% thought it likely that AI would provide medical documentation, 54% to synthesize patient information to reach a diagnosis, 51% to analyze patient information to establish prognosis, and 47% to formulate personalized medication or therapy treatment plans for patients. In total, 36% felt that the benefits of AI would outweigh the risks, 25% felt that the risks would outweigh the benefits, and the rest were uncertain. These findings indicate that the segments of the mental health workforce anticipate that AI will be involved in care provision in some way, and that there are clearly risks and benefits which must be better understood.

The use of AI to support mental health care does come with potential harms [12,20]. For people using AI for mental health support there is the risk of misdiagnosis or misinformation, stemming from AI’s potential for error. There are also questions about the role of empathy in AI systems, although a recent study highlighted that AI can outperform physicians in empathy measures [22]. Data privacy emerges as another salient issue, given the sensitive nature of mental health information and the potential for data breaches or misuse [23]. Biases inherent in nonrepresentative training data can lead to issues of inequity in diagnoses or treatments while the often-opaque decision-making processes of AI systems raise concerns about how complex decisions were made. These potential risks can lead to adverse consequences, and currently, there is limited information regarding the potentially harmful effects of these systems. Consequently, there is a dearth of legislation to safeguard users against such detrimental outcomes [23].

With the widescale popularity of AI technologies such as ChatGPT, it is highly likely that many of these applications are already being used in various ways for mental health care. As society continues to debate the various benefits and risks that this brings, it is critical to understand how and why these technologies are currently being used in the context of mental health care. This study is the first in a series of planned surveys which aims to estimate the current rates of use of AI technology by CMs for mental health and well-being purposes, as well as MHPs for professional purposes, to better understand the scale of use as well as the experienced benefits, harms, and risks associated with its use.

## Methods

### Study Design and Setting

Community members (CMs) and MHPs were invited to complete one of two web-based surveys. The CM survey was advertised to the general population of people aged 16 years and older who reside in Australia. The MHP survey was advertised to MHPs who reside in Australia. The survey was advertised on social media platforms including LinkedIn, Instagram, and Facebook using a snowballing method for 8 weeks between mid-February and mid-April 2024.

### Procedure

The web-based survey was administered using Qualtrics XM (Qualtrics). After accessing the survey link, interested potential participants were screened for eligibility (aged older than 16 years and residing in Australia).

### Measures

The survey included questions regarding the following topics:

- Participant characteristics: demographics for both surveys and clinical service use, and Kessler 10-Item Scale (K10) [24] mental health measure for the CMs survey only. The K10 is scored between 10 and 50. The score ranges are normal (10-19), mild distress (20-24), moderate distress (25-30), and severe distress (>30).
- Technology comfort, attitudes, and use: MHPs and CMs comfort with technology, their attitudes to AI using the AI attitudes scale [25], their interest in AI and their intention for future AI use. The AI attitudes scale is a 4-item scale which asks about general attitudes to AI. Each item is scored between 1 and 10, and the total score is the average score of the 4 items. It has good internal consistency with a Cronbach α of 0.82.
- AI use cases: a number of exemplar AI use cases (see Multimedia Appendix 1) to (1) support CMs mental health and well-being, and (2) to support their MHPs in performing their work duties. CMs and MHPs were presented with a series of potential use cases where AI could assist with specific tasks. Respondents were asked to rate on a scale of 0 to 10 how likely it was that they would use AI for the specific use case.
- Use, experienced benefits and harms: questions for the subset of those who have used AI tools pertaining to their experiences of benefit, harm, and risk.
- Free-text, open-ended responses to both groups about what excites or concerns them regarding the use of AI for mental health care.

The survey is available from the authors upon request.

### Statistical Analyses

Quantitative data were analyzed using descriptive statistics in SPSS (version 22.0; IBM Corp). The sentiment and thematic elements in free-text responses within free-text responses were manually sorted by classifying responses into positive, negative, or neutral categories based on specific keywords and context. Each response was read in its entirety, and sentiments were categorized as positive, negative, or neutral based on the presence of specific keywords and the overall context of the response. Positive sentiments were identified by words such as “hope,” “benefit,” and “optimistic,” among others. Negative sentiments were indicated by terms like “concern,” “risk,” and “fear.” Responses lacking clear sentiment indicators or expressing ambiguous feelings were classified as neutral. Concurrently, thematic analysis [26] was performed to identify and interpret patterns within the data. This involved an iterative process of reading and coding the data, generating initial codes, and collating codes into potential themes. Themes were reviewed and refined through discussions among the research team to ensure they accurately represented the data set.

### Ethical Considerations

Ethical approval for the use of the data was obtained from the University of Melbourne Human Research Ethics Committee (reference 2024-27805-48669-4). This study complied with the Declaration of Helsinki.

## Results

### Sample Characteristics

The final sample consisted of 107 CMs and 86 MHPs. Demographic characteristics of both samples are presented in Table 1. The mean age of both groups was equivalent. The majority of CMs were employed or studying. MHPs tended to have higher levels of formal education than CMs. For MHPs, the majority were either clinical or general psychologists (21/86, 40%). For CMs, the majority had a previous mental health diagnosis or significant difficulties with their mental health or emotional well-being (76/108, 70%), and the majority had also seen a professional for these difficulties (74/108, 69%). The mean K10 score was 22.8 (SD 8.9), indicating mild levels of psychological distress.

**Table 1.** Characteristics of CMs^a^ and MHPs^b^.

Demographic characteristics			CMs (n=108)	MHPs (n=86)
Age (years), mean (SD)			36.9 (16.2)	41.7 (10.9)
**Gender, n (%)**				
	Male		29 (26.9)	27 (31.4)
	Female		71 (65.7)	59 (67)
	Nonbinary, gender diverse, or nonconforming		5 (4.6)	0 (0)
	Prefer not to say		3 (2.8)	0 (0)
**Aboriginal or Torres Strait Islander, n (%)**				
	Yes		4 (3.7)	0 (0)
	No		103 (95.4)	84 (98)
	Prefer not to say		1 (0.9)	2 (2)
**Employment, n (%)**				
	Employed		69 (63.9)	—^c^
	Student		20 (18.5)	—
	Not in the labor force (looking for work, volunteer work, pensioner, or home duties)		19 (17.6)	—
**Income after tax (Aus $; conversion rate of Aus $1=US $0.68 is applicable), n (%)**				
	<$45,000		41 (38.3)	—
	$45,001–$120,000		48 (44.9)	—
	>$120,001		19 (16.8)	—
**Highest level of education, n (%)**				
	High school or equivalent		21 (19.6)	0 (0)
	Technical and further education or associate degree		18 (16.8)	3 (3.5)
	Bachelor degree		29 (27.1)	18 (21)
	Postgraduate diploma or graduate certificate		15 (13.1)	8 (9)
	Masters degree		17 (15.9)	44 (51)
	Doctoral degree or Doctor of Philosophy		8 (7.5)	13 (15)
**Profession, n (%)**				
	Clinical psychologist		—	21 (24)
	General practitioner		—	1 (1)
	Generalist psychologist		—	13 (15)
	Mental health management		—	2 (2)
	Mental health nurse		—	15 (17)
	Occupational therapist		—	4 (5)
	Peer or lived experience worker		—	3 (3.5)
	Psychiatrist or psychiatry registrar		—	4 (5)
	Social worker		—	19 (22)
	Therapist or counselor		—	4 (5)
**Clinical and service use characteristics**				
	**Ever had a previous diagnosis or had significant difficulties with your mental health or emotional well-being? n (%)**			
		Yes	76 (70.4)	—
		No	32 (29.6)	—
	**Have you ever seen a health professional for mental health concerns? n (%)**			
		Yes	74 (68.5)	—
		No	29 (26.9)	—
		Missing	5 (4.6)	—
K10, mean (SD)			22.8 (8.9)	—

a CM: community member.

b MHP: mental health professional.

c Not applicable.

### Technology Comfort, AI Attitudes, and AI Use Intention

In terms of comfort with using digital technology, 79% (68/86) of MHPs and 82% (89/108) of CMs rated themselves as being very comfortable, somewhat comfortable, or comfortable, whereas 22% (19/86) of MHPs and 18% (19/108) of CMs described themselves as being somewhat or very uncomfortable. Table 2 shows responses to the AI attitudes scale. CMs had neutral attitudes and MHPs tended to have more positive attitudes toward AI across all measured dimensions. Tables3  4 show that MHPs also tend to be more interested in using AI and more are more likely to use it in the future for work purposes than CMs are to use AI to manage emotional and mental well-being.

**Table 2.** AI^a^ attitudes scale for CMs^b^ and MHPs^c^.

AI attitudes scale (1=not at all; 10=completely agree)	CMs (n=95), mean (SD)	MHPs (n=82), mean (SD)
I believe that AI will improve my life	5.15 (2.7)	6.62 (2.5)
I believe that AI will improve my work	5.52 (3.0)	6.70 (2.8)
I think I will use AI technology in the future	6.79 (3.0)	7.63 (2.4)
I think AI technology is positive for humanity	5.05 (2.7)	6.00 (2.4)
Average score	5.63 (2.5)	6.74 (2.3)

a AI: artificial intelligence.

b CM: community member.

c MHP: mental health professional.

**Table 3.** Community member interest in the use of artificial intelligence.

Questions		Values
**How interested are you in using AI to support your mental health and emotional well-being? (n=95), n (%)**		
	Not interested at all	26 (27)
	Slightly interested	16 (17)
	Somewhat interested	23 (24)
	Moderately interested	13 (14)
	Extremely interested	17 (18)
**How likely are you to use AI tools in future to support your mental health and emotional well-being? (n=89), n (%)**		
	Very unlikely	15 (17)
	Unlikely	9 (10)
	Somewhat unlikely	9 (10)
	Neither likely no unlikely	13 (15)
	Somewhat likely	21 (24)
	Likely	15 (17)
	Very likely	7 (8)
**How likely are you to use AI for the following (0‐10)? mean (SD)**		
	Mood tracking	5.44 (3)
	Therapeutic chatbots	4.48 (3.3)
	Personalized recommendations	5.72 (3)
	Early detection and monitoring	5.28 (3.2)
	Crisis intervention support	4.47 (3.1)

**Table 4.** Mental health professional interest in the use of artificial intelligence.

Questions		Values
**How interested are you in using AI to assist with tasks in your role as a mental health professional? (n=82), n (%)**		
	Not interested at all	8 (10)
	Slightly interested	9 (11)
	Somewhat interested	13 (16)
	Moderately interested	23 (28)
	Extremely interested	29 (35)
**How likely are you to use these and other AI tools in future to support your work? (n=74), n (%)**		
	Very unlikely	3 (4)
	Unlikely	3 (4)
	Somewhat unlikely	5 (7)
	Neither likely no unlikely	10 (14)
	Somewhat likely	17 (23)
	Likely	13 (18)
	Very likely	23 (31)
**How likely are you to use AI for the following (0‐10), mean (SD)**		
	Assessment and diagnosis	6.12 (3)
	Provide personalized treatment recommendations to clients	6.14 (2.9)
	Track and guide client progress	7.00 (2.6)
	Enhancing client engagement	5.94 (3)
	Administrative assistance	8.16 (2)
	Literature and research analysis	8.07 (2.2)
	Training and simulation	7.43 (2.5)

### AI Use Cases

Table 3 shows that CMs tended to rate their likelihood of using AI for a range of tasks associated with managing their emotional and mental well-being midway between unlikely and likely. The use cases that were most to least popular were (1) providing personal recommendations, (2) mood tracking, (3) detecting early warning signs, (4) use of therapeutic chatbots, and (5) crisis or suicide prevention support. MHPs on the other hand rated themselves as more likely to use AI in use cases across the board. The use cases that were most to least popular were (1) administrative tasks support, (2) synthesizing the latest clinical evidence, (3) training and simulation, (4) tracking consumer progress, (5) personalized recommendations for clients, (6) assisting with assessment and diagnosis, and (7) enhancing consumer engagement with treatment.

### Use of AI

In total, 30 CMs (30/108, 28%) and 37 MHPs (37/86, 43%) reported use of AI in the previous 6 months. Of those, ChatGPT was the most common AI tool used by both CMs (16/30, 52%) and MHPs (20/37, 54%). Table S1 in Multimedia Appendix 1 outlines the reasons respondents provided for using these tools, as well as their experienced benefits, harms, and concerns. Further, 77% (23/30) of CMs and 92% (34/37) of MHPs reported AI to be very beneficial, somewhat beneficial, or beneficial, whereas 10% (3/30) of CMs and 3% (1/37) of MHPs found AI to be very harmful, somewhat harmful, or harmful. CMs mainly used these tools to obtain quick advice when emotionally distressed (18/30, 60%) or as a personal therapist or coach they could converse with to help manage their emotional and mental health (14/30, 47%). The most reported benefits were their availability (20/30, 67%), their low cost compared to therapy (18/30, 60%), and their privacy (16/30, 53%). About half of CMs (16/30, 53%) reported that they did not experience harms or concerns. The rest reported a range of concerns, such as responses being too general or not personalized (11/30, 37%) being unsure where their data was going (11/30, 37%). MHPs primarily used these tools to research mental health topics (24/37, 65%) and to assist with report and letter writing (20/37, 54%). Most reported it being helpful (25/37, 68%) and time saving (25/37, 68%). No harms or concerns were experienced by 49% (18/37); however, the rest reported concerns such as the outputs being too general (12/37, 32%), outputs being inaccurate (10/37, 27%), and being uncertain about the ethics of using these tools for these professional purposes (9/37, 24%).

### Themes and Subthemes of Content Analysis in Free-Text Responses

Respondents were invited to share any concerns or interests they had regarding the use of AI for their specific purposes. A total of 66 responses were received from CMs, and 50 responses were received from MHPs. Among CMs, sentiment was rated as positive in 13 (20%) comments, negative in 17 (26%) comments, and neutral in 38 (58%) comments. Of those with positive sentiment, most were excited about AI making mental health care more accessible and efficient, more personalized, and better integrated with other technologies. The content of their negative sentiment was the lack of human support, errors and misdiagnosis, and ethical or data privacy concerns.

For MHPs of the 50 responses sentiment was rated as positive in 12 (24%) comments, negative in 13 (26%) comments, and neutral in 25 (50%) comments. Positive sentiment themes involved increase in efficiency and therefore increased accessibility of mental health care, as well as advanced diagnostics and treatment outcomes. Negative sentiment comments involved concerns about data governance and security, misuse by clinicians, and regulatory challenges (Tables5  6).

**Table 5.** Analysis of community memebers’ positive and negative sentiment themes on the future of AI^a^ use in mental health care.

Theme		Description	Quote	Community members, n (%)
**Positive sentiment**				13 (20)
	Optimism about accessibility and efficiency	Many are excited about AI’s potential to make mental health care more accessible and efficient, especially in underserved or remote areas.	“AI can provide constant, instant, and affordable support for everyone who needs it.”	12 (92)
	Excitement about technological advancements	Some express a general excitement about the integration of cutting-edge technology in mental health and its potential to revolutionize care.	“There are so many possibilities and it can be revolutionary for mental health diagnosis and treatment.”	10 (77)
	Potential for personalized care	There is enthusiasm for how AI can personalize treatment plans based on individual needs and historical data	“Tailored support for young people to be supported in a way that suits them.”	7 (54)
**Negative sentiment**				17 (26)
	Concerns about lack of human connection	Respondents express concern that AI might not provide the empathetic and nuanced interaction that a human therapist offers	“Lack of human connection increasing the issues that harm mental health in the first place.”	10 (59)
	Ethical and privacy concerns	Concerns regarding the ethical use of AI and data privacy issues are significant, with worries about how sensitive data is handled.	“Concerned about the privacy of therapy sessions when AI is involved.”	9 (53)
	Worries about misdiagnosis or lack of sensitivity	Some fear that AI may not correctly interpret complex human emotions and could lead to misdiagnosis or inappropriate treatment suggestions.	“AI not being able to pick up on serious distress signals that a human would notice.”	8 (47)

a AI: artificial intelligence.

**Table 6.** Analysis of mental health professionals’ positive and negative sentiment themes on the future of AI^a^ use in mental health care for mental health professionals.

Theme		Description	Quote	Mental health professionals, n (%)
**Positive sentiment**				12 (24)
	Technological potential and benefits	Positive views on how AI can enhance the efficiency, accessibility, and quality of mental health care.	“The potential to deliver quality, timely, relevant health care information that allows patient to make more informed choices for their treatment.”	12 (100)
	Technological advancements	Excitement about specific AI technologies that may improve mental health diagnostics and treatment.	“Big data simulations of neural processing; simulations of neurolinguistic indicators of treatment engagement and response, LLM^b^-based mental health co-pilots for both clinicians and patients, no more bloody referral letters!”	5 (42)
**Negative sentiment**				13 (26)
	Risks and misuse	Concerns over potential negative impacts of AI, including risks of misuse by clinicians.	“I have some concern that clinicians may overly rely on AI decisions or outputs that they do not critically analyse the outputs when they make clinical decisions.”	13 (100)
	Ethical and regulatory challenges	Concerns about the lack of adequate ethical guidelines and regulations for AI in health care and the interaction with registered professionals	“ …there need to be enough guardrails to make it safe.” “there is no clinical judgement in AI and the use of this to replace things only clinicians should be practicing.”	5 (38.5)
	Data governance and security	Concerns about how data is managed and protected, focusing on issues like privacy, security, and confidentiality.	“Data governance will be tricky.” “…AI can be used and abused by companies” “data can be shared and sold and then used to manipulate people.” “Confidentiality and only as good as the data in the internet- reflects status quo not creative potential.”	4 (31)

a AI: artificial intelligence.

b LLM: large language model.

## Discussion

### Principal Findings

This study is to our knowledge the first to survey both CMs and MHPs on their patterns of use, experiences, and perceived benefits and harms associated with the application of AI technologies in mental health care. This analysis provides a critical insight into how AI is currently being used to support mental health care from the perspective of CMs and MHPs, which may inform technological development and guide ethical, professional, and policy initiatives.

Attitudes to AI between the groups varied. CMs scored similarly to published community norms on the AI Attitudes Scale [25] (full scale average score 5.63, SD 2.5 vs 5.54, SD 1.78), while MHPs scored significantly higher (full scale average score 6.74, SD 2.3 vs 5.54, SD 1.78). AI use cases for CMs also had lower levels of endorsement than AI use cases for MHPs. Of note, all CM use cases involved scenarios where AI would be used directly to support personal mental health, whereas MHP use cases were split between indirect or administrative professional tasks and direct client mental health support tasks, the former being more likely to be endorsed. Potentially this is due to direct client use cases conceivably carrying more risk, making CMs and MHPs alike wary of using AI in this way. The intended purpose of commercial AI tools is also more aligned with professional support functionalities than direct mental health care applications. The difference highlights a significant area for future development and the necessity of balancing technological advancement with the training and education of both MHPs and CMs in safe use. The difference in use case endorsements also demonstrates that MHPs and CMs experience different pain points in their day-to-day lives. In the challenging context of embedding new technologies into mental health practice, equal consideration should be given to how AI technology can address these pain points for both groups.

Regarding actual use, we found that AI tools, most commonly ChatGPT, were used by around a third of CMs and 40% of MHPs. CMs tended to use these tools to obtain quick mental health advice or to receive emotional support, and nearly half used them as a personal coach or therapist, reporting the benefits being accessibility, privacy, and low-cost. These tools and associated AI techniques have been recognized for their potential to make mental health care more accessible, accurate, and efficient [14,27]. It is important to note, however, that these commercially available AI tools are not intended for such purposes, and as such, may present predictable and unpredictable risks [27]. About half reported experiencing harms or concerns as a result of use, noting that responses were too general, nonpersonalized, inaccurate, or unhelpful. Further, a lack of clarity regarding data security and the ethics of using AI tools in this way was also reported. These kinds of issues can create harm in some cases. For example, Tessa, an integrated rule-based and large language model AI chatbot designed to support patients with eating disorders [28] had to be withdrawn when it started to provide weight loss advice that ran counter to eating disorder clinical guidelines, sparking calls for greater regulatory measures for the safety of these tools in those and other contexts [29]. Survey respondents also expressed concerns regarding the lack of human support and potential for misdiagnosis, which reflects a need for cautious and informed integration of AI into mental health practices, particularly where AI is used to predict illness or risk states [14].

MHPs report using AI tools to support research, administrative, and training tasks, with a substantial number reporting time-saving benefits. Nonetheless, approximately a third of MHPs indicated concerns about the generality and potential inaccuracy of AI outputs, which emphasizes the need for ongoing scrutiny of the quality and application of AI in diverse settings for a wide range of purposes [27,30]. These findings align with the broader discourse in general health care delivery on the integration of AI into care, where efficiency and productivity gains must be balanced with accuracy, reliability, and ethical considerations [20,31].

The expressed concerns by all respondents regarding data governance, security, and the ethical implications of using AI tools in mental health care were notable. As AI technologies continue to advance, it is paramount that data security and ethical use are prioritized to protect both consumers and professionals, and to maintain trust in these tools [20]. As outlined by Luxton [32] a decade ago, psychologists and other mental health care professionals have an essential part to play in the development, evaluation, and ethical use of AI technologies. In a field still grappling to retrofit regulation for non-AI digital health tools, the rapid development of AI health technology and the associated avalanche of personal health data, “blackbox” processing, and data sharing requires swift action to put the necessary safeguard structures in place.

This study has some limitations. First, the online recruitment strategy may have attracted more respondents familiar with technology, although approximately 1 in 5 reported some discomfort with technology use. Second, the relatively small sample size and recruitment method means that the results may not be fully representative of the broader population and therefore limit the generalizability of the findings. This limitation is expected to be addressed in subsequent similar surveys, which will track the acceptability and perceived concerns and issues of using AI in mental health care, over time. Third, the reporting of benefits and harms may be an underestimate as use may have hidden or time-delayed effects. Nevertheless, the findings provide a useful insight into how AI is both currently perceived and experienced by users. Future research could build on these preliminary findings with larger and more diverse samples, potentially through cross-jurisdictional studies that can provide a more comprehensive view of the impact of AI on mental health care.

### Conclusions

Our study underscores the promise and challenges of AI in mental health care. As AI tools evolve, it is essential that they are developed with ethics, inclusivity, accuracy, safety and the genuine needs of end users in mind. This will not only guide technological advancement but also ensure that AI serves as a valuable complement to overwhelmed traditional mental health services, ultimately improving outcomes and efficiencies for all stakeholders involved.

## Supplementary material

10.2196/60589 Multimedia Appendix 1 Tables for artificial intelligence (AI) tool experience for the subset of the community members sample who used AI and AI tool experience for the subset of the mental health professionals sample who used AI tools.

## Abbreviations

AI: artificial intelligence
CM: community member
K10: Kessler 10-Item Scale
MHP: mental health professional
