# Supplementary material for: Use of AI in Mental Health Care: Community and Mental Health Professionals Survey
Source: JMIR Ment Health. 2024 Oct 11;11:e60589. doi: 10.2196/60589 (PMC11488652; doi:10.2196/60589)
Supplement: Multimedia Appendix 1 [file mental-v11-e60589-s001.docx]

**Table S1.** AI tool experience for the subset of the community members sample who used AI tools. AI: artificial intelligence.

| **Community members** | **n (%)** |
| --- | --- |
| **What do you use these tools for?*** |  |
| Getting quick advice when emotionally distressed | 18/30 (60%) |
| As a personal therapist or coach I could converse with to help me manage my emotional and mental health | 14/30 (46.7%) |
| Provide insights into my mental health and emotions | 11/30 (36.7%) |
| Learning more about mental health | 10/30 (33.3%) |
| Getting advice about how to help others | 8/30 (26.7%) |
| Getting support when having thoughts of harming yourself | 4/30 (13.3%) |
| Information about how where to get help | 3/30 (10.0%) |
| **What were the benefits of using these tools?*** |  |
| It was always or easily available. | 20/30 (66.7%) |
| It was cheaper than talking to a therapist | 18/30 (60.0%) |
| It was private, I didn’t have to talk to a professional person | 16/30 (53.3%) |
| It was non-judgmental | 13/30 (43.3%) |
| It was helpful and improved my emotional or mental health | 9/30 (30.0%) |
| It was empathetic | 9/30 (30.0%) |
| It was accurate in the advice it provided | 4/30 (13.3%) |
| **What harms, concerns or inconveniences were experienced?*** |  |
| No harms experienced | 16/30 (53.3%) |
| Its responses were not personalised to me and were too general | 11/30 (36.7%) |
| Not sure where my personal data was going | 11/30 (36.7%) |
| It was unhelpful | 7/30 (23.3%) |
| Wasn’t sure if I could trust the advice | 4/30 (13.3%) |
| It wasted my time | 3/30 (10%) |
| It was inaccurate and gave me the wrong advice | 3/30 (10%) |
| It made me feel emotionally or mentally worse than I was to begin with | 3/30 (10%) |
| It appeared to be biased | 2/30 (6.7%) |
| Didn’t trust the company that provided it | 2/30 (6.7%) |
| It said it couldn’t help me | 1/30 (3.3%) |
| **How would you rate the effectiveness of these tools to support your mental health and emotional wellbeing?** |  |
| Very beneficial | 1/30 (3.3%) |
| Beneficial | 11/30 (36.7%) |
| Somewhat beneficial | 11/30 (36.7%) |
| Neither beneficial no harmful | 4/30 (13.3%) |
| Somewhat harmful | 1/30 (3.3%) |
| Harmful | 1/30 (3.3%) |
| Very harmful | 1/30 (3.3%) |

*multiple responses allowed.

**Table S2.** AI tool experience for the subset of the MHPs sample who used AI tools. AI: artificial intelligence; MHP: mental health professional.

| **MHPs** |  |
| --- | --- |
| **What do you use these tools for?*** |  |
| Re1searching or find out more information about a mental health topic | 24/37 (64.9%) |
| Assist with letter or report writing | 20/37 (54.1%) |
| Synthesising information to arrive at a diagnosis | 9/37 (24.3%) |
| Assist with writing clinical progress notes | 6/37 (16.2%) |
| Assisting with assessment | 5/37 (13.5%) |
| Medication or treatment planning and strategies | 4/37 (10.8%) |
| Responding therapeutically to clients online (e.g. email, webchat) | 4/37 (10.8%) |
| **What were the benefits of using these tools?*** |  |
| It was helpful | 25/37 (67.6%) |
| It saved me a lot of time | 25/37 (67.6%) |
| It was always available | 16/37 (43.2%) |
| It produced better output than I could have done myself | 14/37 (37.8%) |
| It was accurate | 11/37 (29.7%) |
| It provided me with information I wouldn’t have otherwise obtained | 11/37 (29.7%) |
| No benefit | 1/37 (2.7%0 |
| **What harms, concerns or inconveniences were experienced?*** |  |
| No harms experienced | 18/37 (48.6%) |
| Its outputs were too general | 12/37 (32.4%) |
| It was inaccurate | 10/37 (27.0%) |
| Uncertain about the ethics of using the technology in this way | 9/37 (24.3%) |
| Wasn’t sure if I could trust the advice | 8/37 (21.6%) |
| Not sure where my / my client’s data or personal information was going | 7/37 (18.9%) |
| It produced worse outputs than I could have produced myself | 6/37 (16.2%) |
| It was unhelpful | 4/37 (10.8%) |
| It appeared to be biased | 2/37 (5.4%) |
| Didn’t trust the company that provided it | 5/37 (37%) |
| **How would you rate the effectiveness of these tools in supporting you in your work?** |  |
| Very beneficial | 14/37 (37.8%) |
| Beneficial | 7/37 (18.9%) |
| Somewhat beneficial | 13/37 (35.1%) |
| Neither beneficial no harmful | 2/37 (5.4%) |
| Somewhat harmful | 1/37 (2.7%) |
| Harmful | 0/37 (0%) |
| Very harmful | 0/37 (0%) |

*multiple responses allowed.
